# Deletion of Mgr2p Affects the Gating Behavior of the TIM23 Complex

**DOI:** 10.3389/fphys.2018.01960

**Published:** 2019-01-15

**Authors:** Oygul Mirzalieva, Shinhye Jeon, Kevin Damri, Ruth Hartke, Layla Drwesh, Keren Demishtein-Zohary, Abdussalam Azem, Cory D. Dunn, Pablo M. Peixoto

**Affiliations:** ^1^Department of Natural Sciences, Baruch College, The City University of New York, New York City, NY, United States; ^2^Department of Biochemistry and Molecular Biology, The George S. Wise Faculty of Life Sciences, Tel Aviv University, Tel Aviv, Israel; ^3^Institute of Biotechnology, Helsinki Institute of Life Science, University of Helsinki, Helsinki, Finland; ^4^Department of Molecular Biology and Genetics, Koç University, Istanbul, Turkey; ^5^MCD Program, The Graduate Center, The City University of New York, New York City, NY, United States

**Keywords:** Mgr2, TIM23, mitochondria, reactive oxygen species, protein import

## Abstract

The TIM23 complex is a hub for translocation of preproteins into or across the mitochondrial inner membrane. This dual sorting mechanism is currently being investigated, and in yeast appears to be regulated by a recently discovered subunit, the Mgr2 protein. Deletion of Mgr2p has been found to delay protein translocation into the matrix and accumulation in the inner membrane. This result and other findings suggested that Mgr2p controls the lateral release of inner membrane proteins harboring a stop-transfer signal that follows an N-terminal amino acid signal. However, the mechanism of lateral release is unknown. Here, we used patch clamp electrophysiology to investigate the role of Mgr2p on the channel activity of TIM23. Deletion of Mgr2p decreased normal channel frequency and increased occurrence of a residual TIM23 activity. The residual channel lacked gating transitions but remained sensitive to synthetic import signal peptides. Similarly, a G145L mutation in Tim23p displaced Mgr2p from the import complex leading to gating impairment. These results suggest that Mgr2p regulates the gating behavior of the TIM23 channel.

## Introduction

The biogenesis of most proteins in the mitochondrial matrix and the inner membrane relies on the function of the TIM23 complex. The list of imported proteins includes regulators of core metabolic functions (Sass et al., 2003; Danczak-Pazdrowska et al., 2012), mitochondrial dynamics (Herlan et al., 2004; Ishihara et al., 2006), and of mitophagy (Jin et al., 2010), which underlies the importance of TIM23 to mitochondrial function. The core of the TIM23 complex contains a pore that is essential for protein insertion into or translocation across the inner membrane. The pore is permeable to proteins harboring a structurally conserved amino-terminal acid domain or “presequence” which warrants translocation into the matrix, unless that domain is followed by a hydrophobic “stop-transfer” amino acid sequence (Bohnert et al., 2010). The gating of this protein-selective pore has been extensively studied in patch clamp experiments on isolated mitochondrial membranes. Patch clamping was also instrumental to functional dissection the main TIM23 components (Lohret et al., 1997; Kinnally et al., 2000; Martinez-Caballero et al., 2007a). There are five membrane-spanning TIM23 subunits: Tim23p and Tim17p are thought to be the pore components (Martinez-Caballero et al., 2007a); Tim50p is the main presequence receptor and a putative structural scaffold that links TIM23 to the outer membrane import complex (TOM) (Mokranjac et al., 2009); Tim21p is a transient subunit that has been shown to interact with TOM or with respiratory complexes (Chacinska et al., 2005); Mgr2p/Romo1 bridges TIM23 to the presequence translocase-associated motor (PAM) and is thought to regulate the lateral release of proteins harboring stop-transfer sequences. Interestingly, there is contrasting evidence that Tim21p either prevents or facilitates the coupling between TIM23 and PAM (Gebert et al., 2012; Ieva et al., 2014). Moreover, the protein Pam18p reportedly regulates this coupling by displacing Mgr2p and promoting anterograde import of matrix-targeted proteins (Schendzielorz et al., 2018).

The conserved Mgr2 protein was firstly identified in a genetic screen searching for genes required for survival of yeast cells lacking mitochondrial DNA (Dunn et al., 2006). Several studies on the mammalian variant, Romo1 (reactive oxygen species modulator 1) suggested its involvement in regulation of mitochondrial morphology and apoptosis (Chung et al., 2006; Zhao et al., 2009; Kim et al., 2010; Norton et al., 2014). More recent studies showed that Mgr2p could be co-purified with the TIM23 complex. In mitochondria lacking Mgr2p, the TIM23 complex was devoid of Tim21p and was no longer associated with the PAM complex (Gebert et al., 2012). As a consequence, substrates normally directed to the matrix accumulated at the inner membrane (Ieva et al., 2014). On the other hand, overexpression of Mgr2p enhanced sorting of proteins into the matrix. Taken together, these results supported the interpretation that Mgr2p regulates the dual sorting function of TIM23. However, it is unknown whether Mgr2p impacts the channel activity of TIM23. Here, we address this question using patch clamp electrophysiology. Our results indicate that Mgr2p deletion impacts the channel gating behavior, which may impinge upon its role in the dual sorting function of the TIM23 complex.

## Materials and Methods

### Yeast Strains and Isolation of Mitochondrial Inner Membranes

*Saccharomyces cerevisiae WT* (*MATa his3Δ1 leu2Δ0 met15Δ0 ura3Δ0; BY4741*) and *mgr2Δ* (*MATa his3Δ1 leu2Δ0 met15Δ0 ura3Δ0 mgr2ΔkanMX4; EUROSCARF Y02154*) mutant strains generated in the S288C background were grown at 32°C, 225 rpm in YPLac medium to OD600 of 1.0. The *TIM23* and *tim23-G145L* mutant strains (Demishtein-Zohary et al., 2015) were grown in YPLac to OD 1.0 at 24°C, 120 rpm.

Respiring mitochondrial inner membranes were extracted from 2 g of yeast pellets by differential centrifugation and treated in a French press. After purification, the inner membranes were reconstituted in proteoliposomes (Peixoto et al., 2007). Briefly, 5 μg membranes and 1 mg small phosphatidylcholine (Sigma-Aldrich) liposomes were mixed in 5 mM HEPES (N-2-hydroxyethylpiperazine-N′-2-ethanesulfonic acid, pH 7.4), and dotted on a glass slide. The dots were dehydrated for ∼3 h and then rehydrated overnight with 150 mM KCl and 5 mM HEPES, pH 7.4, at 4°C. Once reconstituted, the membranes were harvested with 0.5 mL of the same media and stored in small aliquots at -80°C.

### Pull-Down Assays

Mitochondrial pellets (300 μg) from *TIM23* and *tim23-G145L* strains were processed for pull down assays as previously described (Ieva et al., 2014). Briefly, mitochondrial extracts were incubated with 30 μL Ni^2+^–nitrilotriacetic acid–agarose beads (Hylabs) and 250 μL pull-down buffer in 0.05% digitonin. After 45 min incubation in a rolling shaker at 40°C, the mitochondrial samples were centrifuged at 1000 *g* for 1 min, unbound fractions (supernatant) were collected, and the bound fractions (pellet) were washed three times with 250 μl of pull-down buffer supplemented with 0.05% digitonin. These fractions were analyzed by immunoblotting with anti-Tim17p, anti-Tim23p, anti-Tim50p, and anti-Mgr2p antibodies.

### Patch Clamping

Patch-clamp procedures and analyses were described elsewhere (Peixoto et al., 2007). The patch solution was 150 mM KCl and 5 mM HEPES, pH 7.4 both in the pipette and in the bath (symmetrical conditions). TIM23 channel identity was confirmed by bath perfusion with 5 μM yCoxIV_(1-13)_ presequence. Currents with abnormal gating properties but with yCoxIV_(1-13)_ sensitivity were ascribed as “residual” Tim23 channels. Currents were recorded from at least three individual inner membrane preparations from each yeast strain. Voltage clamp was performed with the excised configuration of the patch-clamp technique using an Axopatch 200 or Dagan 3900 amplifier. Currents were low-pass filtered at 2 kHz and digitized with a sampling rate of 5 kHz using a Digidata 1322A digitizer and Clampex 8.2 software (Axon Instruments). Clampfit 8.2 (Axon Instruments) and WinEDR 2.3.3 (Strathclyde Electrophysiological Software; courtesy of J. Dempster, University of Strathclyde, United Kingdom) were used for analysis of channel activity.

## Results and Discussion

The multi-subunit TIM23 complex is the main pathway for protein import into the mitochondrial inner membrane and the matrix (Schulz et al., 2015). Despite intensive characterization over the previous decades, new TIM23 subunits and interaction partners continue to emerge. More recently, co-precipitation studies have identified the Mgr2 subunit, which is believed to serve as a “gatekeeper” for protein sorting in the yeast mitochondrial inner membrane. Based on *in vitro* protein import experiments into yeast mitochondrial fractions either lacking or overexpressing Mgr2p, it was suggested that this new subunit regulates the lateral release of preproteins into the inner membrane. Mgr2p was shown to crosslink with the preproteins in transit, suggesting its close proximity to the translocation pore (Ieva et al., 2014). However, the exact mechanism of protein translocation is unknown.

### Effect of Mgr2p Removal Upon TIM23 Channel Activity

**FIGURE 1:**
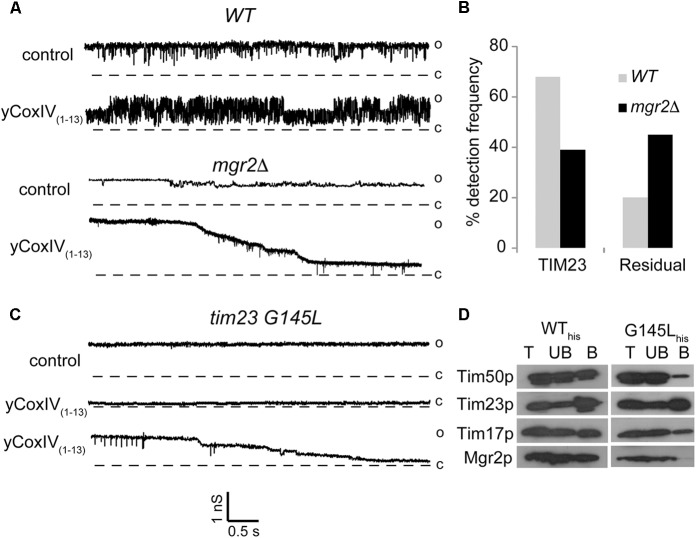
Effect of Mgr2 deletion on the channel activity of TIM23. **(A–C)** Representative patch clamp current traces show TIM23 channel activity recorded from indicated mitochondrial inner membrane preparations before and after addition of 5 μM presequence peptide yCoxIV_(1-13)_. The vertical and horizontal scale bars indicate conductance in nanosiemens and time in seconds, respectively. Open “o” and closed “c” states are indicated to the right of the current traces. **(B)** Bar histograms of normal and residual TIM23 channel frequencies in *WT* and *mgr2Δ* mitochondria. Data are average from *n* > 50 recordings per condition. **(D)** Immunoblotting following a pull-down assay shows the presence of the indicated TIM23 components in total (T), unbound (UB) and bound B fractions from mitochondria containing wild type (WT_his_) or mutant (G145L_his_) Tim23 protein.

We examined the impact of Mgr2p deletion on the channel activity of TIM23 using patch clamp electrophysiology. Mgr2p deletion was confirmed by immunoblot and immunofluorescence (Supplementary Figure S1) and the integrity of the remaining TIM23 core complex was reported elsewhere (Gebert et al., 2012). Figure 1A shows sample current traces representative of normal TIM23 conductance rapidly gating between open, semi-open, and closed levels (Martinez-Caballero et al., 2007b). Albeit similar to that of TIM22, this gating behavior is sensitive to synthetic presequences like yCoxIV_(1-13)_ (Peixoto et al., 2007). Therefore, presequence sensitivity can be used to ascertain the identity of TIM23-like channel currents in inner membrane preparations. Wild type mitochondrial inner membranes displayed normal TIM23 channels in 69.8 % of the patches (*n* = 50, Figure 1B). Conversely, this frequency dropped to 39 % when analyzing *mgr2Δ* mitochondria. This was accompanied by an increase in residual channel activities that were still sensitive to the synthetic presequences (Figures 1A,B). Aside from increasing normal TIM23 activity, it has been previously demonstrated that yCoxIV_(1-13)_ can be imported by respiring mitochondria (Martinez-Caballero et al., 2007b). This was confirmed routinely in our preparations, and Mgr2p elimination did not impair yCoxIV_(1-13)_ accumulation (Supplementary Figure S2). Interestingly, *mgr2Δ* mitochondria displayed disparate sensitivity to synthetic presequences: rather than a reversible increase in activity, the residual channels underwent a stepwise and irreversible closure (not shown).

In order to independently verify these results, we resorted to a yeast strain harboring a G145L mutation in the second transmembrane helix of the Tim23 protein. This mutation caused loss of the Mgr2 subunit from the TIM23 complex, as shown in pull down experiments using the Tim23 protein as bait (Figure 1D). Patch clamp analysis showed residual TIM23 activity, as seen in the *mgr2Δ* membranes (Figure 1C). However, there was an even more dramatic decrease in normal channel frequency (∼16%, not shown), which at closer examination of the pull-down experiments may reflect partial loss of the channel subunit Tim17p as well as Tim50p from the *tim23-G145L* strain. In accordance, it has been previously demonstrated that depletion of Tim17p renders current traces with no gating transitions and altered sensitivity to presequence signal peptides (Martinez-Caballero et al., 2007a). A more recent study showed that removal of an intramolecular disulfide bond facing the inner membrane space of Tim17p (between cysteines 10 and 77) caused similar gating impairment (Schendzielorz et al., 2018). Based on examination of deletion strains, it was proposed that the bond is catalyzed by the intermembrane space oxidoreductase Erv1p. Another candidate promoting disulfide bond formation might be Mgr2p given that its deletion caused gating impairment akin to that observed in the *tim17* mutants. Future studies will investigate this possibility, as well as the ability of Mgr2 overexpression to reverse the gating impairment in the Tim23p mutants.

Despite some similarity to the Tim17p depletion studies, our results do not support a structural role of Mgr2p within the TIM23 pore. Instead, the presence of both normal and residual activities indicates that Mgr2p depletion affects the stability of the TIM23 core complex. It is unclear if Mgr2p transiently interacts with the TIM23 complex *in vivo*. If this were the case, the patch clamp data could indicate that the insertion mechanism involves synchronized disassembly of TIM23 during protein import, thus entrapping preproteins in the inner membrane. Future studies will address this possibility by evaluating native TIM23 activity and integrity in the presence of synthetic peptides harboring both an N-terminal and a stop-transfer presequence.

## Author Contributions

OM performed the patch clamp experiments, mitochondrial isolations, and immunohistochemistry. SJ and RH performed the ROS measurements. KD performed the yCoxIV accumulation and membrane potential measurements. LD and KD-Z performed the pull down experiments. AA generated the Tim23G145L strain, supervised experiments, and revised the manuscript. CD generated the Mgr2p antibody, performed the immunoblot in Supplementary Figure S1, and revised the manuscript. PP performed the patch clamp, experimental design and supervision, and manuscript writing.

## Conflict of Interest Statement

The authors declare that the research was conducted in the absence of any commercial or financial relationships that could be construed as a potential conflict of interest.
