# Supplementary figures and images for: Deletion of Mgr2p Affects the Gating Behavior of the TIM23 Complex
Source: Front Physiol. 2019 Jan 15;9:1960. doi: 10.3389/fphys.2018.01960 (PMC6340964; doi:10.3389/fphys.2018.01960)

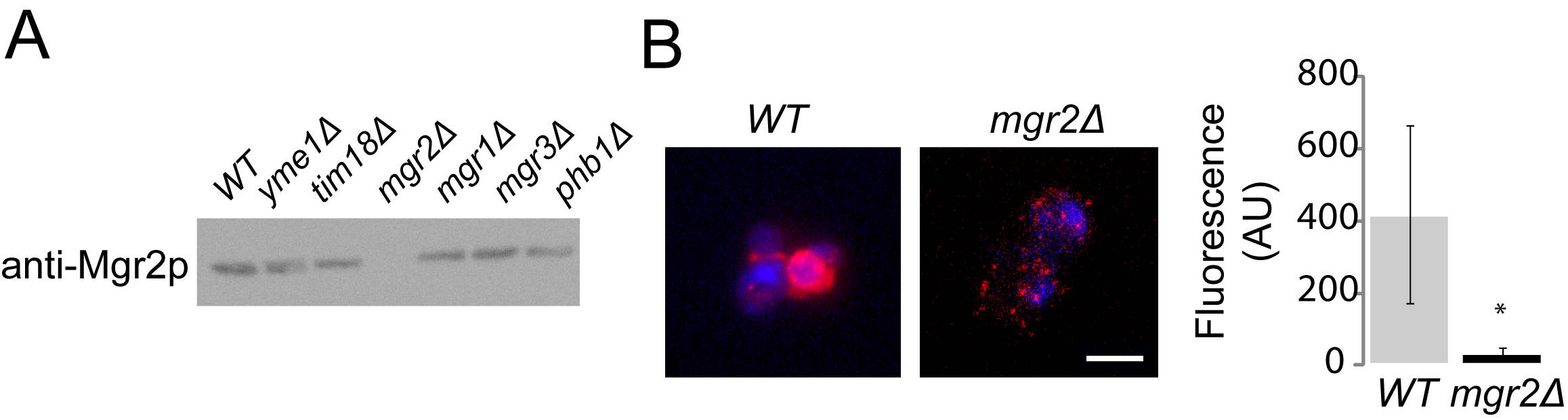

Supplement: Supplementary file 1 [file Image_1.tif]

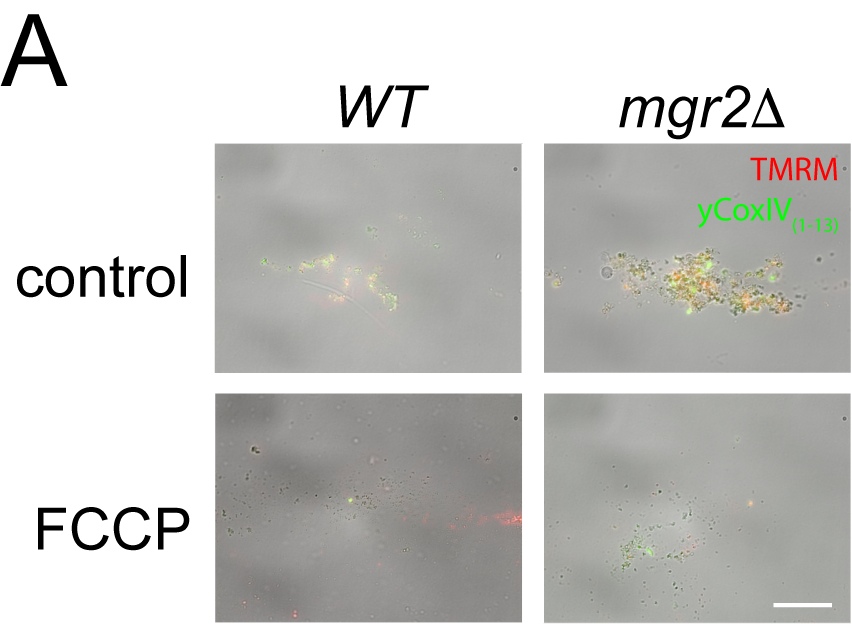

Supplement: Supplementary file 2 [file Image_2.tif]
