# Supplementary material for: Deletion of Mgr2p Affects the Gating Behavior of the TIM23 Complex
Source: Front Physiol. 2019 Jan 15;9:1960. doi: 10.3389/fphys.2018.01960 (PMC6340964; doi:10.3389/fphys.2018.01960)
Supplement: Supplementary file 3 [file Table_1.docx]

**Supplemental Materials and Methods**

*Yeast Strains*

The list below summarizes *S. cerevisiae* strains used in this study. All but the *Tim23-G145L* and *WT* strains are available in the European Saccharomyces Cerevisiae Archive for Functial Analysis (EUROSCARF).

CDD1 - WT (BY4741)

CDD7 - yme1∆ (EUROSCARF Y07144)

CDD9 - tim18∆ (EUROSCARF Y01593)

CDD11 - mgr2∆ (EUROSCARF Y02154)

CDD13 - mgr1∆ (EUROSCARF Y03451)

CDD15 - mgr3∆ (EUROSCARF Y06555)

CDD17 - phb1∆ (EUROSCARF Y04762)

*Mgr2p antibody production and analysis of Mgr2 expression in yeast*

The anti Mgr2p IgG was purified from rabbit antiserum inoculated with synthetic peptides (_3_PLPQNYAQQQPSNWDKFKC_20_ and _76_CIRSDSESSPMSHPNL_91_, respectively). The antibody production and ELISA testing were outsourced through StorkBio LTD (Estonia). For analysis of Mgr2p deletion and antibody specificity, total protein extracts (50 μg) were separated in 13.5% MES-SDS polyacrylamide electrophoresis gels and blotted onto nitrocellulose membranes following standard procedures. Protein blots were blocked in 1x Tris Buffered Saline (TBS) containing 0.2% Triton X-100 and 1 % fat free cow milk and then incubated with anti-Mgr2p (1:500) followed by detection using horseradish peroxidase-conjugated antibodies and chemiluminescence-based detection (ECL, Molecular Probes).

Mgr2p expression was also analyzed by immunofluorescence. In brief, yeast cells were grown as described in the methods to OD600 1.0 and permeabilized spheroplasts (1mL) were obtained after treatment with lyticase (2 units/mL, Fischer Scientific) and Triton X-100 (0.1% in TBS). Yeast cells (10 μL) were dotted onto poly-L-lysine coated glass coverslips (Fischer) and incubated with anti-Mgr2p antibody (1:100) in blocking TBS solution for 1 h at room temperature. Cells where then incubated in blocking TBS three times for 5 min prior to addition of anti rabbit TexasRed-IgG (1:1000, Thermofisher) and nuclear dye Hoescht 33342 (10 μm, Molecular Probes). Fluorescence images were acquired using a CoolSNAP HQ2 monochrome camera on a Nikon Eclipse TE2000-E microscope equipped with a 100 X Fluor objective, automated shutters, a motorized stage for autofocusing, and a heating chamber.

*Presequence accumulation in isolated mitochondria*

Amino-terminal tagging of the synthetic peptide yCoxIV_(1-13)_ (MLSLRQSIRFFKY, Thermofisher) with fluorescein isothiocyanate (FITC) was performed according to manufacturer instructions (Molecular Probes). Accumulation of FITC-labelled yCoxIV_(1-13)_ into isolated (12.5 μg, 1 mg/mL) mitochondria was carried out in import buffer (0.6 M mannitol-sucrose, 25 mM KCl, 10 mM MgCl_2_, 2 mM KPO_4_, 0.5 mM EDTA, 2 mM ATP, 2 mM NADH, 50 mM HEPES–KOH, pH 7.4) by incubation at 30 °C for 10 min. FCCP (1 μM) was included for negative controls. Mitochondria were pelleted by centrifugation at 14,000 g for 10 min, washed three times, and re-suspended in import buffer prior to imaging on a NIKON TE2000 microscope as above.

**Supplemental Figure Legends**

**Figure S1: Analysis of Mgr2 expression.** A) Immunoblots of total protein extracts from the indicated *S. cerevisiae* strains labeled with anti-Mgr2p antibody (1:500). B) Representative merged fluorescence microscopy images of wild type (*WT*) and mutant (*mgr2∆*) yeast strains labeled with purified rabbit anti-Mgr2p antibody (1:100) and nuclear dye Hoescht 3334 (10 μM). The red fluorescence in *mgr2∆* was similar to negative controls in absence of the anti-Mgr2p antibody (not shown). Scale Bar is 10 μm. The bar histogram (right) shows average Mgr2p fluorescence (± SD, n = 5).

**Figure S2: Accumulation of presequence peptide into isolated mitochondria.** A) Merged bright field and fluorescence microscopy images of mitochondria isolated from the indicated strains. Red and green fluorescence represent TMRM and yCoxIV_(1-13)_, respectively. Accumulation of FITC-labelled yCoxIV_(1-13)_ into isolated (12.5 μg, 1 mg/mL) mitochondria was carried out in import buffer (0.6 M mannitol-sucrose, 25 mM KCl, 10 mM MgCl_2_, 2 mM KPO_4_, 0.5 mM EDTA, 2 mM ATP, 2 mM NADH, 50 mM HEPES–KOH, pH 7.4) by incubation at 30 °C for 10 min. FCCP (1 μM) was included for negative controls. Mitochondria were pelleted by centrifugation at 14,000 g for 10 min, washed three times, and re-suspended in import buffer prior to imaging. Scale bar is 10 μm.
